# Genomic Reference Resource for African Cattle: Genome Sequences and High-Density Array Variants

**DOI:** 10.1038/s41597-024-03589-2

**Published:** 2024-07-19

**Authors:** Abdulfatai Tijjani, Sumaya Kambal, Endashaw Terefe, Regina Njeru, Moses Ogugo, Gideon Ndambuki, Ayao Missohou, Amadou Traore, Bashir Salim, Chukwunonso Ezeasor, Claire D’andre H., Emmanuel T. Obishakin, Boubacar Diallo, Essodina Talaki, Issaka Y. Abdoukarim, Oyekanmi Nash, Richard Osei-Amponsah, Simeone Ravaorimanana, Youssouf Issa, Tsadkan Zegeye, Christopher Mukasa, Christian Tiambo, James G. D. Prendergast, Stephen J. Kemp, Jianlin Han, Karen Marshall, Olivier Hanotte

**Affiliations:** 1 Centre for Tropical Livestock Genetics and Health (CTLGH), ILRI Ethiopia, P.O. Box 5689, Addis Ababa, Ethiopia; 2 The Jackson Laboratory, 600 Main Street, Bar Harbor, Maine 04609 USA; 3 Department of Genetics and Animal Breeding, Faculty of Animal Production, University of Khartoum, Khartoum, Sudan; 4 Department of Animal Science, College of Agriculture and Environmental Sciences, Arsi University, Asella, Ethiopia; 5 International Livestock Research Institute, P.O. Box 30709, Nairobi, 00100 Kenya; 6 Ecole Inter-Etats des Sciences et Médecine Vétérinaires (EISMV), Dakar, Sénégal; 7 Institut de l’Environnement et de Recherches Agricoles (INERA), Ouagadougou, Burkina Faso; 8 Faculty of Veterinary Medicine, University of Khartoum, Khartoum, Sudan; 9 Camel Research Center, King Faisal University, Al-Ahsa, Saudi Arabia; 10 Department of Veterinary Pathology and Microbiology, University of Nigeria, Nsukka, Enugu State Nigeria; 11 Rwanda Agricultural and Animal Resources Development Board, Kigali, Rwanda; 12 Biotechnology Division, National Veterinary Research Institute, Vom, Plateau State Nigeria; 13 Central Vétérinaire de Diagnostic (LCVD), Conakry, Guinea; 14 École Supérieure d’Agronomie de l’Université de Lomé, Lomé, Togo; 15 Laboratoire de Biotechnologie Animale et de Technologie des Viandes, Abomey-Calavi, Benin; 16 Centre for Genomics Research and Innovation, NABDA, Abuja, Nigeria; 17 Department of Animal Science, College of Basic and Applied Sciences, University of Ghana, Legon, Ghana; 18 Ministère de l’Agriculture, de l’Elevage et de la Pêche, Antananarivo, Madagascar; 19 Institut National supérieur des Sciences et Techniques d’Abéché-INSTA/Tchad, Abéché, Chad; 20 Mekelle Agricultural Research Center, Tigray Agricultural Research Institute, Mekelle, Ethiopia; 21 National Animal Genetic Resources Centre and Data Bank (NAGRC&DB), Entebbe, Uganda; 22 Centre for Tropical Livestock Genetics and Health (CTLGH), Roslin Institute, University of Edinburgh, Easter Bush Campus, Midlothian, EH25 9RG UK; 23 CAAS-ILRI Joint Laboratory on Livestock and Forage Genetic Resources, Institute of Animal Science, Chinese Academy of Agricultural Sciences (CAAS), Beijing, China; 24 Yazhouwan National Laboratory, No. 8 Huanjin Road, Yazhou, Sanya, 572024 Hainan P. R. China; 25 Cells, Organism and Molecular Genetics, School of Life Sciences, University of Nottingham, Nottingham, UK

**Keywords:** Genetic variation, Evolutionary genetics

## Abstract

The diversity in genome resources is fundamental to designing genomic strategies for local breed improvement and utilisation. These resources also support gene discovery and enhance our understanding of the mechanisms of resilience with applications beyond local breeds. Here, we report the genome sequences of 555 cattle (208 of which comprise new data) and high-density (HD) array genotyping of 1,082 samples (537 new samples) from indigenous African cattle populations. The new sequences have an average genome coverage of ~30X, three times higher than the average (~10X) of the over 300 sequences already in the public domain. Following variant quality checks, we identified approximately 32.3 million sequence variants and 661,943 HD autosomal variants mapped to the Bos taurus reference genome (ARS-UCD1.2). The new datasets were generated as part of the Centre for Tropical Livestock Genetics and Health (CTLGH) Genomic Reference Resource for African Cattle (GRRFAC) initiative, which aspires to facilitate the generation of this livestock resource and hopes for its utilisation for complete indigenous breed characterisation and sustainable global livestock improvement.

## Background & Summary

The indigenous African cattle breeds represent a valuable genetic resource that underpins sustainable productivity and resilience in the face of environmental challenges, including the impacts of climate change^1^. Their ability to thrive in diverse and often challenging environments makes them vital assets for local communities, the broader agricultural sector, and potentially offers novel genetic solutions to livestock systems beyond Africa. Recent works have highlighted the unique taurine, indicine (zebu), and taurine × indicine genome admixture of indigenous African cattle^2–11^. In addition, Kambal *et al*.^12^ summarized several candidate genes under positive selection in sub-Saharan African cattle breeds for environmental adaptation, including disease resilience, heat tolerance, and adaptation to high altitudes. Talenti *et al*.^13^ reported the first global cattle graph genome, including two African breeds (N’Dama and Ankole).

To fully harness the potential of these indigenous cattle breeds for sustainable agriculture and climate resilience, there is an urgent need to invest in genomic research and the development of comprehensive genomic resources. By enhancing our understanding of the genetic basis of productivity and adaptive traits in African cattle, we can support targeted breeding strategies, informed conservation efforts, and the development of innovative solutions to address environmental challenges and secure food and livelihood security for local communities^14^.

Despite significant advancements in NGS (Next-Generation Sequencing), high-throughput technologies, and increasing affordability and accessibility, indigenous African cattle genetic data in the global cattle genomic resources remain limited. Only a few studies have characterised African cattle breeds’ full autosomal genome diversity. For example, one of the most recent and comprehensive genome sequence studies, conducted by Kim *et al*.^6^, focused on characterizing 15 predominantly African zebu breeds, most of which originate from Eastern Africa. Tijjani *et al*.^9^ reported the autosomal genome characterization of five Sudanese cattle breeds at a country level. Likewise, Terefe *et al*.^10^ and Zegeye *et al*.^11^ analysed the diversity of Ethiopian breeds, and Paguem *et al*.^15^ reported the characterization of five indigenous breeds from Cameroon.

However, no cattle genome sequence has been reported for most sub-Saharan African countries. Consequently, the lack of comprehensive genomic data hinders the complete characterization of African cattle diversity and the development of genomic tools for precision breeding and rapid genetic improvement of livestock. These tools, including informed crossbreeding, marker-assisted selection, genomic selection, and genome editing, could significantly enhance production traits and adaptive performance in African cattle populations and have potential applications in temperate systems. Addressing the diversity gap in genomic data representation and conducting further research will unlock the potential for more informed and efficient cattle breeding on the continent. Moreover, collating a set of whole genome sequences of representative African cattle populations could be utilized as a high-quality reference panel for accurate and cost-effective genotype imputation of a larger sample size^16^.

The Centre for Tropical Livestock Genetics and Health (CTLGH) is a partnership between The Roslin Institute, the Royal (Dick) School of Veterinary Studies at the University of Edinburgh, Scotland’s Rural College, and the International Livestock Research Institute. The mission of CTLGH is to improve the livelihoods of people who depend on livestock in tropical regions. To achieve this, CTLGH conducts research projects that study the diversity of tropical livestock and identify the genetic factors contributing to their production traits and ability to adapt to environmental pressures. CTLGH is working with African national partners to conduct this research on cattle, with the support of the African Union Inter-African Bureau for Animal Resources (AU-IBAR) through the Genomic Reference Resource for African Cattle (GRRFAC) initiative.

**Fig. 1:**
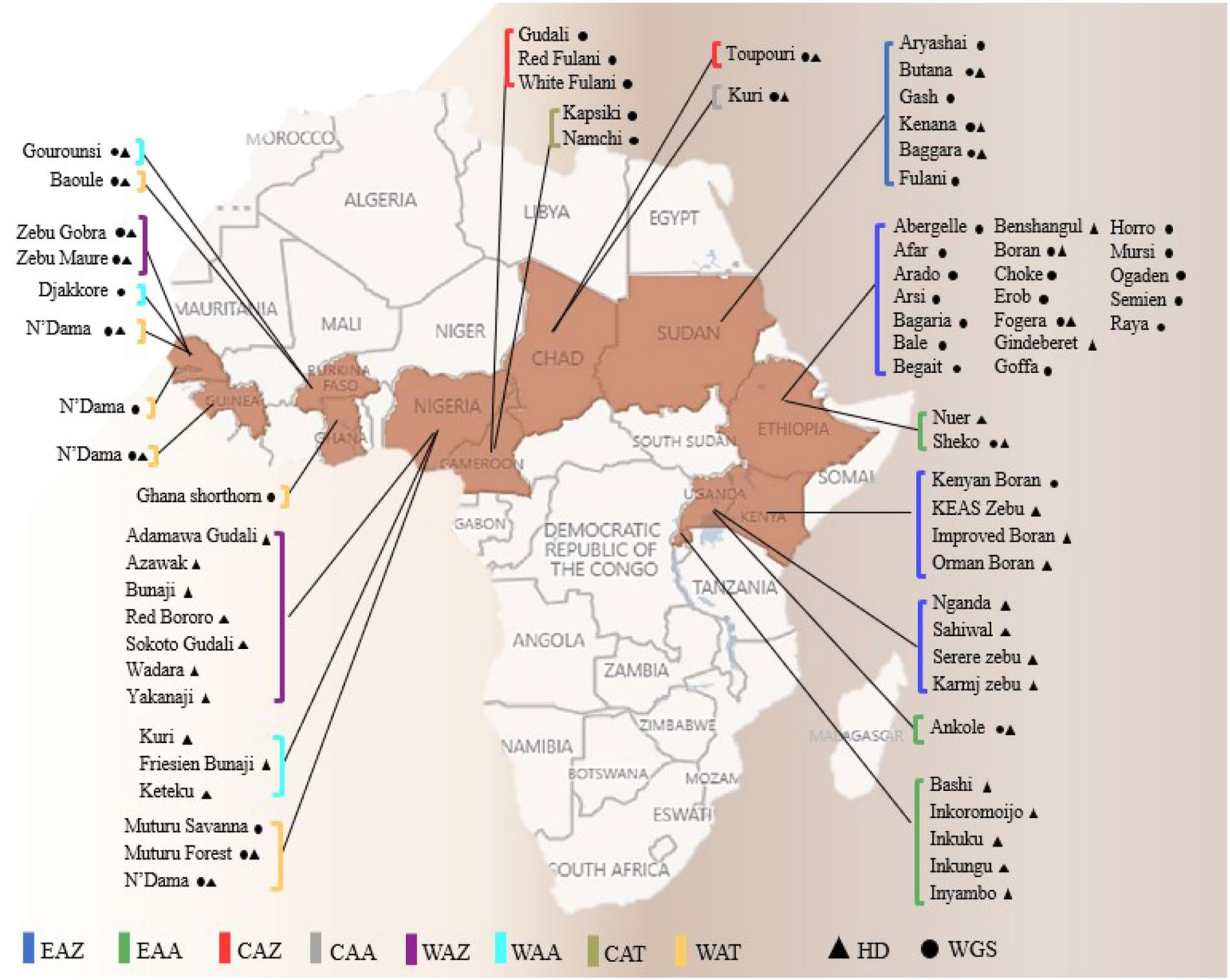
The African continent map shows the sampling countries for high-density genotyping (HD, triangle) and whole-genome sequencing (WGS, circle). East African zebu (EAZ), East African admixed (EAA), Central African zebu (CAZ), Central African Admixed (CAA), West African zebu (WAZ), West African admixed (WAA), Central African taurine (CAT), and West African taurine (WAT).

In the initial phase, African National partners from 13 countries contributed over 1000 cattle blood samples following the Access and Benefit Sharing (ABS) permits granted by the relevant countries’ authorities. The samples came mainly from countries in East Africa (Ethiopia, Kenya, Sudan, and Rwanda), Central Africa (Chad and Cameroon), and West Africa (Benin, Burkina Faso, Ghana, Guinea, Nigeria, Senegal, and Togo), and comprised (based on phenotypic characteristics) several African zebu, African taurine, and African admixed (e.g., Sanga) breeds (Fig. 1). We genotyped 537 individuals from these samples using the Illumina® BovineHD DNA Analysis Kit and performed whole genome sequencing of 208 individuals (Supplementary Tables 1, 2). The dataset comprised more than 18 terabytes of paired-end sequences generated on the Illumina HiSeq X platform with individual sequence coverage ranging from 15.2X to 56.4X at an average of 30X.

Here, we report our progress in collating whole-genome sequences and HD genotyping data of indigenous African cattle. Some endangered indigenous African breeds with distinct phenotypes (see Mwai *et al*.^17^ for a review), including the Toupouri and Kuri from Chad, Orma Boran from Kenya, Forest Muturu subtype from Nigeria, and different cattle populations from Rwanda, were included for the first time (genome sequences or HD genotyping data). Also, our dataset comprised novel sequences of different trypanotolerant N’Dama populations from Senegal, Guinea, and Nigeria (Supplementary Tables 1–4).

## Methods

### New sample collection and genomic DNA isolation

Standardized sampling protocols, approved by ILRI’s research ethics committee (approval number: IACUC-RC2016-18), were endorsed and applied by the African project partners. An experienced scientist from partner countries oversaw the sampling exercise, collecting biological, pedigree, and phenotypic data, including photographs of animals, using the Open Data Kit (ODK) tool. Approximately 10 ml of whole blood was duplicated from each animal into EDTA tubes. All blood samples were sent to the ILRI-Nairobi biorepository and laboratory, where genomic DNA (gDNA) was extracted. The sample metadata can be requested from ILRI *via* the project dataset portal (https://data.ilri.org/portal/dataset?q=grrfac).

Per the manufacturer’s instruction, DNA was extracted using the PureLink Genomic DNA Mini Kit (Cat no: K182002, Invitrogen Inc., Carlsbad, CA, USA). First, 200 µl of frozen-thawed blood was added to 200 μl of PBS and mixed thoroughly. Next, 20 μl proteinase K and 20 μl RNase A were added to the mixture, then briefly vortexed and incubated at room temperature for 2 minutes. Next, 200 μl PureLink® Genomic Lysis/Binding Buffer was added and mixed by vortexing, followed by incubation for 10 min at 55 °C on a heating block. Then, 200 µl of absolute ethanol was added, mixed thoroughly, and briefly centrifuged. Six hundred and forty µl of the sample was then transferred to the PureLink® Spin Column and centrifuged at 10,000 × g for 1 minute at room temperature. The columns were washed with 500 µl of the kit wash buffer 1 and 2. The DNA was immediately quantified using the Qubit 4 Fluorometer Invitrogen Qubit dsDNA HS Assay according to the standard kit protocol, indicating the DNA concentrations in the starting sample before dilution. DNA was then diluted in Tris-EDTA buffer to a 50 ng/μl concentration. At room temperature, a minimum of 50 μl was shipped to the Edinburgh Genomics (https://genomics.ed.ac.uk/) for sequencing.

### Library preparation and DNA sequencing

Next-generation sequencing libraries were prepared using the Illumina SeqLab-specific TruSeq PCR-Free High Throughput library preparation kit in conjunction with the Hamilton MicroLab STAR and Clarity LIMS X (4.2) Edition. The gDNA samples were normalised to the concentration and volume required for the Illumina TruSeq PCR-Free library preparation kit and then sheared to a 450 bp mean insert size using the Covaris LE220 focused ultrasonicator. The inserts were blunt-ended, A-tailed, size selected, and TruSeq-adapters ligated onto the ends.

The insert size for each library was evaluated using the Caliper GX Touch with an HT DNA 1k/12 K/HI SENS LabChip and HT DNA HI SENS Reagent Kit to ensure that the mean fragment sizes fall between 300 bp and 800 bp. The concentration of each library was calculated using the Roche LightCycler 480 and a Kapa Illumina Library Quantification Kit and Standards to ensure that the concentration of each library was between 1.1 nmol and 8 nmol.

The libraries were normalised to 1.5 nmol and then denatured for clustering and sequencing at 300 pmol using THE Hamilton MicroLab STAR with Genologics Clarity LIMS X (4.2) Edition. The libraries were clustered onto the HiSeqX Flow cell v2.5 on cBot2s and the clustered flow cell was transferred to the HiSeq X Ten System for sequencing using the HiSeq X Ten Reagent kit v2.5.

### Sequence alignment and variant calling

**Fig. 2:**
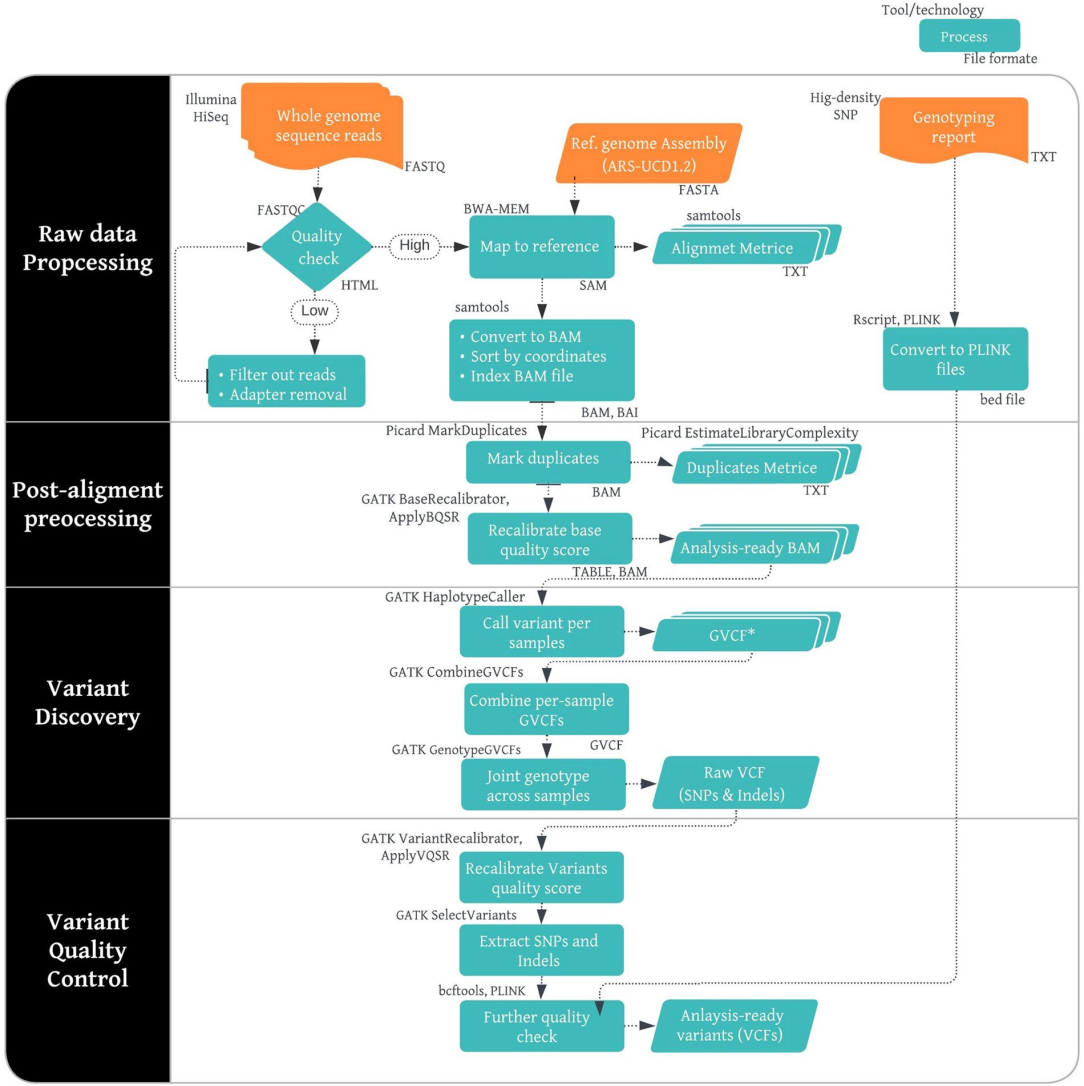
An overview of the workflow for sequence mapping and variant discovery pipeline. The pipeline follows the Broad Institute’s recommended Genome Analysis Tool Kit best practices for germline short variant discovery.

In addition to the 208 new genomes generated *via* the GRRFAC initiative, we retrieved 347 African cattle genomes from public databases. Most of these were from previous ILRI and University of Nottingham collaborative studies with African partners (Supplementary Tables 2, 3). The fastq files containing the cattle genome sequence data were processed (as outlined in the workflow shown in Fig. 2) to produce a single VCF file.

The paired-end reads from individual genomes were aligned to the *Bos taurus* reference genome ARS-UCD1.2^18^, using the bwa mem mode of Burrows-Wheeler Alignment tool (BWA) of either version 0.7.13-r1126 or 0.7.17^19^. We used the customized version of the reference assembly (ARS-UCD1.2_Btau5.0.1Y), in which the *Y* chromosome from a previous bovine assemble (Btau5.0.1Y) was incorporated into the ARS-UCD1.2 assembly, as provided by the 1000 Bull Genomes project (1KBGP)^20^. We achieved an average alignment rate of 99%. The samtools version 1.11^21^ was used to sort and index the initial alignment file, and the Picard tools version 1.119 (http://broadinstitute.github.io/picard) was used to mark duplicate reads. The variant discovery protocol followed the Broad Institute’s recommended GATK Toolkit best practices for germline short variant discovery (https://software.broadinstitute.org/gatk/best-practices/)^22^, as illustrated in Fig. 2, which led to the detection of single nucleotide polymorphisms (SNPs) and insertions/deletions (InDels). Specifically, base quality score recalibration (BQSR) was performed on the sorted alignment file in Binary Alignment (Bam) format before writing out the final reads. The GATK *HaplotypeCaller* module was used to calculate genotype likelihoods for individual genomes to produce a genomic variant calling format (gVCF) that contained a record of every position of the examined region in the bovine genome. Next, we merged the individual genomic gVCF files using the *CombineGVCFs* module before performing joint genotyping of all 555 gVCF files simultaneously for variant discovery using the GATK *GenotypeGVCF* module.

Variant quality score recalibration (VQSR) was then applied to filter raw autosomal variants using truth and training datasets provided by the 1KBGP. Variants (SNPs and InDels) above 90.0% of the truth sensitivity level from VQSR were retained. Using the BCFtools version 1.11 (http://samtools.github.io/bcftools/bcftools.html), we filtered the detected SNPs by removing multiallelic and monomorphic SNPs. Also, the detected InDels were further filtered to exclude variants with more than 50 bp. Finally, approximately 32.3 million SNPs and 4.5 million InDels were obtained from a joint variant call across the 555 African cattle genomes.

### Genotyping and quality control

Of the over 1000 samples contributed by African National partners, genomic DNA was extracted from 537 individuals following the above protocol, and the samples were genotyped using the Illumina® BovineHD DNA Analysis Kit (Illumina, San Diego, CA), which comprised about 777,962 SNPs. Using PLINK version 1.9^23^, the datasets received in eight cohorts were processed and converted to the PLINK binary formats and then merged. Subsequently, these samples were combined with publicly available HD datasets of African cattle^3,4,16,24^ to produce a final dataset of 1082 African samples. Following the approach of Riggio *et al*.^16^ and using coordinates from the NAGRP Shared Data Repository as described by Schnabel^25^, we lift over the UMD3.1 bovine assembly positions of the HD array to the cattle reference ARS-UCD1.2 assembly^18^. We fixed allele strand inconsistencies and converted the combined genotype data to a VCF file.

## Data Records

The new sequencing data of indigenous African cattle (in FASTQ format) for 176 samples can be accessed from the NCBI Sequence Read Archive (SRA) under accession numbers **PRJNA853448**^26^ **and PRJNA1075356**^27^. The sequencing data of the remaining 32 samples is deposited at the European Nucleotide Archive (ENA) under accession numbers **PRJEB39924**^28^ and **PRJEB39353**^29^. The variant data for this study have been deposited in the European Variation Archive (EVA)^30^ at EMBL-EBI under accession numbers **PRJEB74565**^31^ and **PRJEB74477**^32^.

## Technical Validation

### Sequence data metrics and quality control of variants

**Fig. 3:**
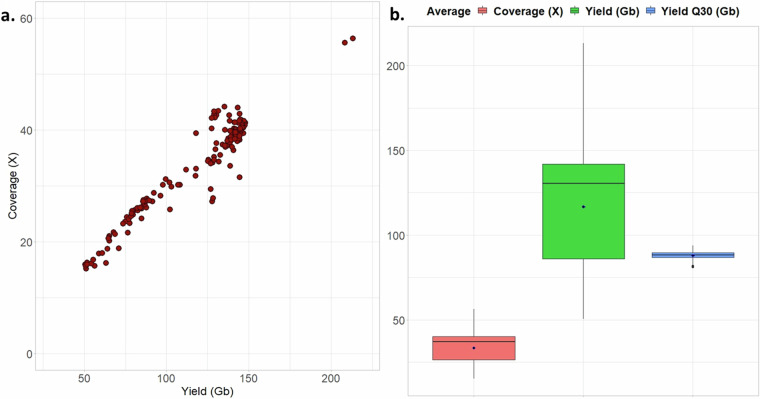
The distribution of coverage and estimated sequence yield for 208 new genomes of indigenous African cattle.

An average of 116 Gigabase (Gb) of raw sequencing data were generated for each of the new genomes (range of 50.9 Gb to 213.3 Gb sequencing yield), for which an average of 88 Gb (75%) had a minimum Phred scaled quality score of 30, an indication of the expected base calling accuracy of 99.9 percent. The sequence coverages for the new genomes ranged from 15.2X to 56.4X (Fig. 3).

**Fig. 4:**
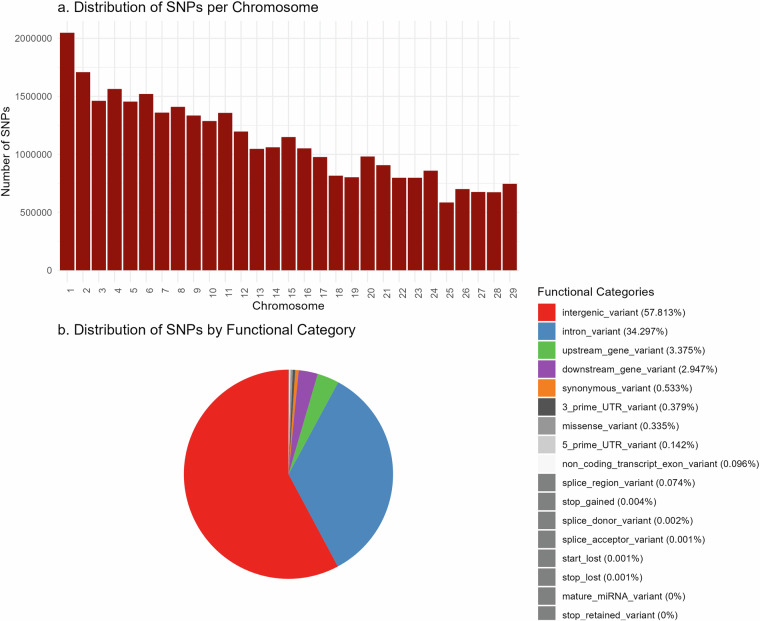
The distribution of detected SNPs in indigenous African cattle per *Bos taurus* autosomal chromosomes and along functional categories. (**a**) Distribution of SNPs per Chromosome. (**b**) Distribution of SNPs by Functional Category.

The distribution of detected SNPs in indigenous African cattle along the *Bos taurus* autosomal chromosomes in our sequence variant dataset is shown in Fig. 4a. The distribution along functional categories reveal that about 58% and 34% of detected variants are in the intergenic regions and introns, respectively, whereas variants within the exons comprise less than 1% (Fig. 4b). The ratio of transitions to transversion (Ti/Tv) is a quality measure for next-generation sequencing. It is based on the theoretical assumption that the probability of a purine nucleotide conversion to another purine, or pyrimidine to pyrimidine, in a mutational process is only half of a transversion^33,34^. A Ti/Tv ratio 2.36 was calculated for our 32.3 M SNPs after VQSR filtration, indicating good quality of our SNP calling.

**Fig. 5:**
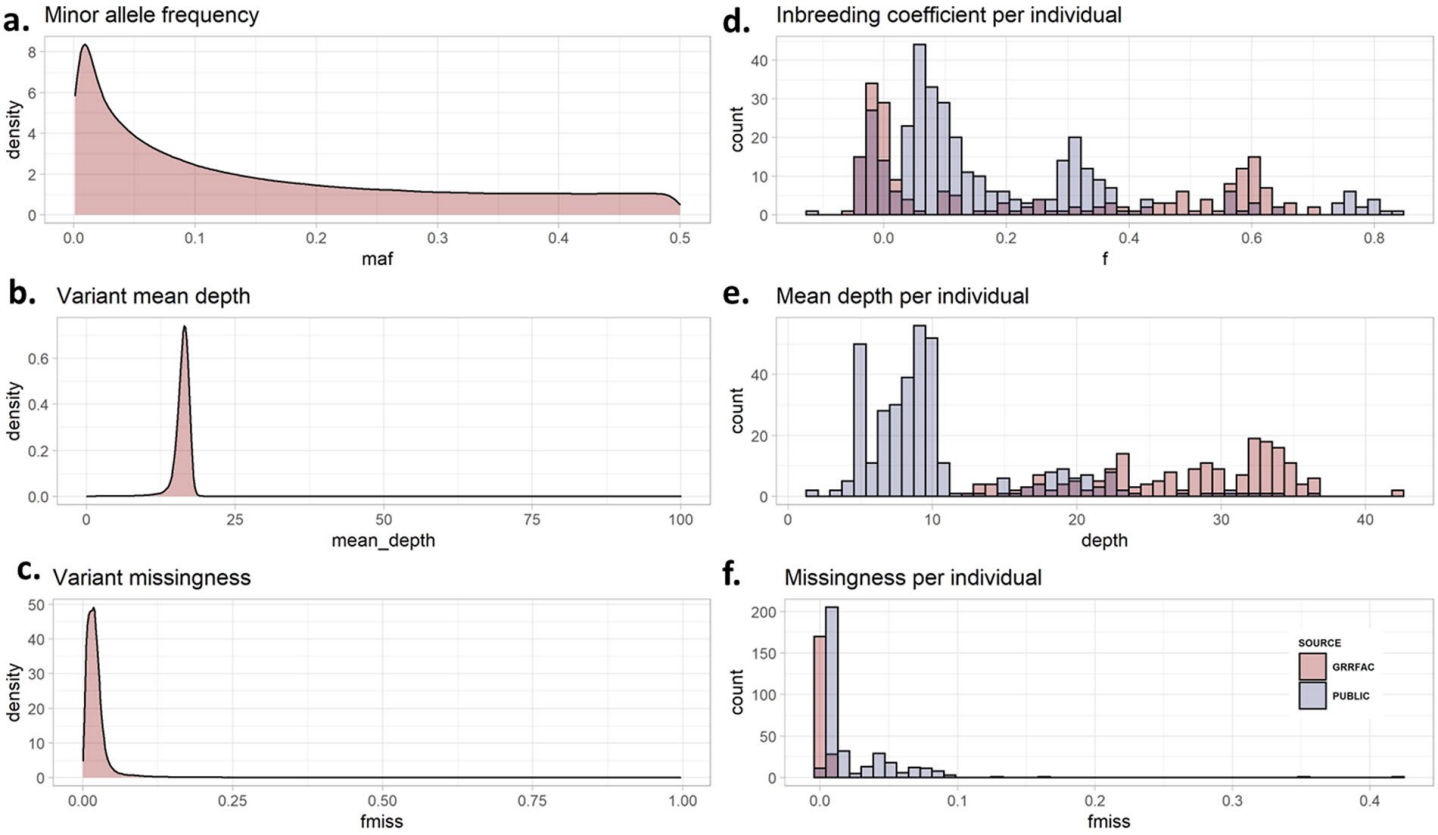
Sequencing quality metrics across 555 (208 new and 347 publicly available) genomes of indigenous African cattle. (**a**) Minor allele frequency (**b**) Inbreeding coefficient per individual (**c**) Variant mean depth (**d**) Mean depth per individual (**e**) Variant missingness (**f**) Missingness per individual.

Proper QC measures are essential to minimize the inclusion of erroneous variants and enhance the integrity of downstream analyses. Hence, we further evaluated the detected variants of some quality control metrics across all collated 555 African cattle samples using a subset of the detected autosomal SNP data. We restricted the dataset to a subset of approximately 2.2 million SNPs, obtained by thinning the initial 32.3 M SNPs to 1 SNP in every 1 kb. Using VCFtools^35^, we evaluated variant-based statistics such as the distribution of minor allele frequency (MAF), variant mean depth, and variant missingness (Fig. 5a–c).

Understanding the distribution of allele frequencies in a genomic dataset is essential as the choice of MAF thresholds can dramatically affect population genetic inference^36^. The distribution of MAF in our variant data shows a predominance of rare variants (Fig. 5a). Low MAF alleles may only occur in a relatively small number of individuals or are unreliable calls^36,37^. However, in diverse populations, such as indigenous African cattle, rare variants can be crucial in shaping complex traits and genetic diversity within livestock populations. This diversity is essential for adaptation to changing environments and overall population health. Rare variants might also be important reservoirs of genetic potential for future breeding programs. Hence, just as in humans, caution must be taken when determining the MAF threshold in livestock genetic studies, where applicable. As such, we did not filter the new datasets based on MAF.

The variant mean depth at a specific site is the number of reads mapped to this position. Figure 5b shows a mean read depth of approximately 20X across all individuals in our dataset. Outlier regions with high coverage likely reflect mapping errors or repetitive regions. In Fig. 5c, we examine the proportion of individuals that lack genotype at each variant position (variant missingness). Indeed, most sites have almost no missing data, and we can be quite conservative in setting the threshold for missing sites.

Also, we calculated sample-level variant statistics, such as the inbreeding coefficient, mean depth, and the proportions of missing data per individual (Fig. 5d–f), which are critical for ensuring the reliability, accuracy, and robustness of genomic data. These statistics provide valuable insights into the quality of each sample genetic information and the variants detected within them. These individual-based estimates also enable us to compare the GRRFAC project data to those already in the public. We estimated the level of inbreeding among all cattle samples (Fig. 5d). The inbreeding coefficient, often denoted as (“f”), ranges from 0 to 1, with values closer to 1 indicating higher levels of inbreeding. Regarding individual genotypes, higher levels of inbreeding signify that an individual is more likely to have inherited identical alleles from both parents for a particular gene than expected under the Hardy-Weinberg equilibrium. Hence, such an individual is more likely to be homozygous at various gene loci, leading to a decrease in heterozygosity. While some individuals in the collated datasets were highly inbred (f > 0.5, n = 92), the majority (n = 353) had inbreeding coefficients below 0.2 (Fig. 5d).

In line with the sequence coverage estimates in Fig. 3, individual sequencing depth estimated from detected variants ranges from <3X to ~40X across the 555 cattle samples (Fig. 5e), most of the GRRFAC samples have higher sequencing depth >20X (dark red coloured) compared to most of the public data, which are below 10X depth, with some individual depths as low as 3X (Fig. 5e).

Lastly, the genome-wide proportions of missing sequence variants per individual genome ranged from 0.01 to 0.43. None of our project samples had more than 5% missing SNPs, but 17 samples from the public data had more than 20% missing variants (Fig. 5f and Supplementary Table 5). These samples were excluded from the final datasets. Likewise, two samples from the HD SNP data had high proportions of missing variants above 20% and were excluded (Supplementary Table 6).

### Analysis of sample relatedness and principal component analysis

We assessed pairwise individual relatedness among all cattle samples based on the genome sequence and HD SNP array datasets using the*–relatedness2 option* of VCFtools^35^. This analysis is based on the KING method, for which kinship coefficients (‘Phi’) are >0.354 for duplicate samples and monozygotic twins, [0.177–0.354] for first-generation relatives, [0.0884–0.177] for second-generation relatives, [0.0442–0.0884] for third-generation relatives, and <0.0442 for unrelated samples^38^. We detected seven pairs of duplicates and 12 pairs of first-degree relatives among the samples in the sequence data, including two Butana and two Gash samples from Sudan that were likely sample mix-ups as previously reported by Tijjani *et al*.^9^ (see Supplementary Table 5). From the HD SNP data, we identified 17 highly related samples (refer to Supplementary Table 6). Both datasets had two samples, each with high proportions of variant missingness.

**Fig. 6:**
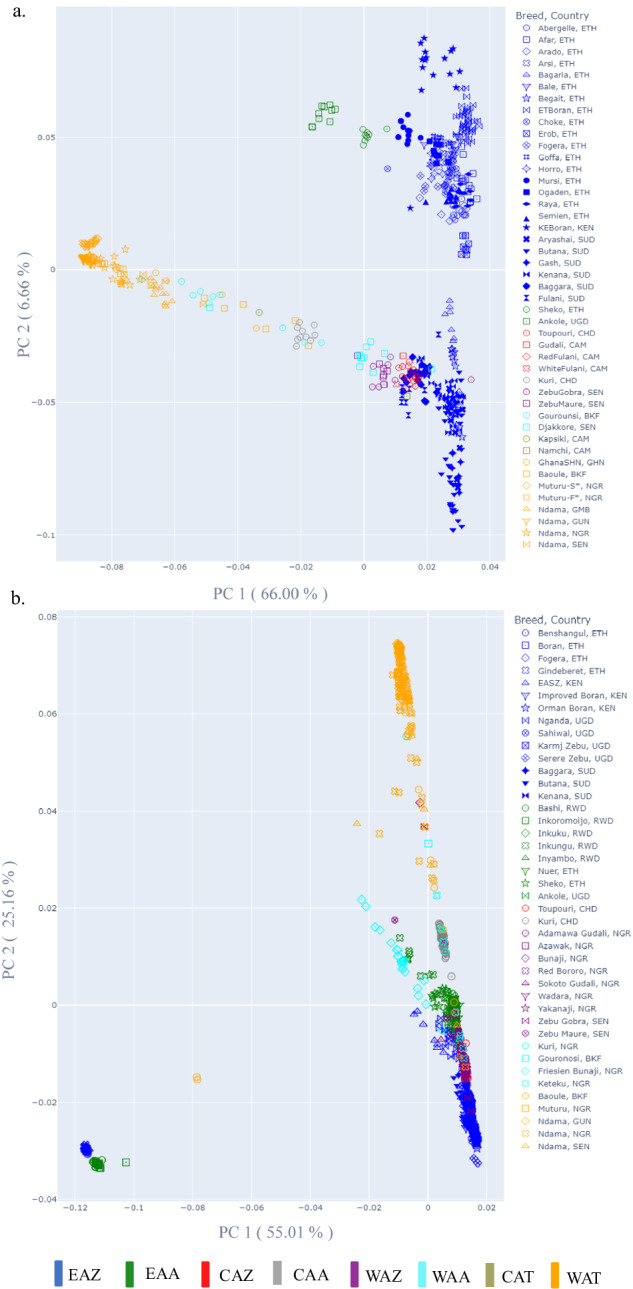
Population structure of indigenous African cattle depicted through principal component analysis (PC 1 against PC 2). (**a**) PCA of 532 samples (41 African cattle breeds), based on the whole-genome sequences, and (**b**) PCA of 1063 samples (39 African cattle breeds) based on the HD SNP genotypes. Six groups of breeds are denoted by different colours and abbreviated as follows: East African zebu (EAZ), East African admixed (EAA), Centre African zebu (CAZ), West African zebu (WAZ), Centre and West African admixed (CWZ), Centre and West African taurine (CWT). Countries abbreviation: Ethiopia (ETH), Kenya (KEN), Uganda (UGD), Rwanda (RWD), Sudan (SUD), Chad (CHD), Cameroon (CAM), Senegal (SEN), Burkina Faso (BKF), Ghana (GHN), Nigeria (NGR), Gambia (GMB), Guinea (GUN). * Savana (S) Muturu and Forest (F) Muturu.

Finally, using PLINK, the cattle samples that failed the QC criteria were excluded from the two datasets. Principal components analysis confirmed that individual cattle samples clustered with their respective populations. In Fig. 6, each plot of the first two principal components separated the African zebu cluster from the Central and West African taurine cohorts. Moreover, with the sequence data, we observed a further separation across the zebu, with the East African zebu of Ethiopia separated from those of Sudan origin and the Central and West African zebu (Fig. 6a). In contrast, the HD SNP data did not reveal this level of resolution (Fig. 6b). We observed outliers in breeds such as Benshangul, Gindeberet, Fogera (blue), Nuer and Sheko (green), and Baoule (yellow).

### Supplementary information

Supplementary Table 1

Supplementary Table 2

Supplementary Table 3

Supplementary Table 4

Supplementary Table 5

Supplementary Table 6

## Data Availability

No custom code was used to process the current dataset described here.
